# Fu-Fang-Jin-Qian-Cao herbal granules protect against the calcium oxalate-induced renal EMT by inhibiting the TGF-β/smad pathway

**DOI:** 10.1080/13880209.2020.1844241

**Published:** 2020-11-15

**Authors:** Wen-Rui Liu, Hong-Tao Lu, Ting-Ting Zhao, Jia-Rong Ding, Ya-Chen Si, Wei Chen, Jie-Bin Hou, Song-Yan Gao, Xin Dong, Bing Yu, Zhi-Yong Guo, Jian-Rao Lu

**Affiliations:** a Department of Nephrology, Seventh People's Hospital Affiliated to Shanghai University of Traditional Chinese Medicine, Shanghai, China; b Changhai Hospital, Second Military Medical University, Shanghai, China; c Department of Naval Medicine, Second Military Medical University, Shanghai, China; d Department of Geriatric Nephrology, The Second Medical Centre, Chinese PLA General Hospital, Beijing, China; e Institute of Translational Medicine, Shanghai University, Shanghai, China; f Department of Cell Biology, Second Military Medical University, Shanghai, China

**Keywords:** Oxalate crystals, renal fibrosis, traditional Chinese medicine

## Abstract

**Context:**

Nephrolithiasis is a major public health problem worldwide and Fu-Fang-Jin-Qian-Cao granules (FFJQC) is a traditional Chinese herbal formula that is used to treat nephrolithiasis. The main component of nephrolithiasis is calcium oxalate (CaOx) and the epithelial-mesenchymal transition (EMT) shown to play a crucial role in CaOx-induced kidney injury. However, the mechanism underlying the therapeutic effect of FFJQC on the CaOx-induced renal EMT is unknown.

**Objective:**

This study explores the therapeutic benefits and mechanism of FFJQC in oxalate-induced kidney injury.

**Materials and methods:**

60 male C57BL/6 mice were used in this experiment and divided into 6 groups. A mouse kidney stone model was created by intraperitoneal injection of glyoxylate at a dose of 100 mg/kg for 6 days. The standardized FFJQC was used to treat mouse crystal kidney injury by gavage at 1.35 and 2.7 g/kg, respectively. Western blotting and immunostaining for E-cadherin, cytokeratin 18 (CK18), vimentin, smooth muscle α-actin (α-SMA) and transforming growth factor β (TGF-β)/Smad pathway were conducted on renal tissues.

**Results:**

Following CaOx-induced kidney injury, the levels of E-cadherin and CK18 in kidney decreased, while vimentin and α-SMA levels increased. The FFJQC treatment increased the levels of E-cadherin and CK18 and decreased vimentin and α-SMA levels in varying degrees. What’s more, the FFJQC reduced the expression of CaOx-induced fibrosis marker collagen II.

**Conclusion:**

FFJQC alleviated the CaOx-induced renal EMT and fibrosis by regulating TGF-β/smad pathway. Therefore, the FFJQC is an important traditional Chinese medicine for the treatment of CaOx-induced renal injury and fibrosis.

## Introduction

Nephrolithiasis is a common disorder associated with painful kidney stone episodes, and increased risk of chronic kidney disease (CKD), as well as end-stage renal disease (ESRD) (El-Zoghby et al. 2012; Shoag et al. 2014). The prevalence of nephrolithiasis varies in different countries. In Western countries, the prevalence of nephrolithiasis ranges from 0.1 to 14.8% of the population (Romero et al. 2010), whereas in China, the prevalence is 5.8% of the population (Zeng et al. 2017). But the incidence of nephrolithiasis has been reported to be increasing globally in recent years (Kirkali et al. 2015).

Calcium oxalate (CaOx) crystals are the major crystalline constituents of kidney stones (Xu et al. 2017) which stimulate local inflammation and injury, particularly near the tubules cells. Crystalline deposition induced epithelial-mesenchymal transition (EMT) has been observed both *in vivo* (Hu et al. 2015) and *in vitro* (Zhang, Yuan, et al. 2017). Renal fibrosis markers have also been detected in CaOx model (Convento et al. 2017), which would help to interpret the association between renal function decline and kidney stone cases without urinary tract obstruction. Therefore, the CaOx crystals induced EMT plays crucial roles in urolithiasis related renal injury.

During EMT progression, cells acquire mesenchymal characteristics, along with the downregulation of epithelial markers, such as E-cadherin, cytokeratins, and the tight junction protein zonula occludens-1 (ZO-1), resulting in the disintegration and loss of cell-cell contacts. In contrast, cells undergoing the EMT upregulate mesenchymal markers, such as vimentin and α-SMA (Zeisberg and Neilson 2009). Overall, the transforming growth factor β1 (TGF-β1) signalling axis plays a crucial role in the progression of the EMT and renal fibrosis (Meng et al. 2015; Chen et al. 2018). According to previous studies, CaOx could stimulate the TGF-β1 production in renal tubular cells (Convento et al. 2017), and activate the PI3K/Akt signalling pathway (Wang et al. 2019). The functional complex of TGF-β family receptors at the cell surface consists of type I (TGF-β RI) and type II (TGF-β RII) receptors. The binding of TGF-β1 to a heterotetramer composed of TGF-β RI and TGF-β RII induces a conformational change in TGF-β RII. TGF-β RII is autophosphorylated and then phosphorylates TGF-β RI to activate downstream receptor-associated Smad (R. Smads: Smad2 and Smad3) signalling molecules. R. Smads and Smad4 enter the nucleus to regulate gene transcription along with other transcriptional regulatory molecules, while Smad6 and Smad7 inhibit other Smads (Derynck and Zhang 2003).Therefore, the molecular mechanism of possible strategies designed to prevent the CaOx induced renal EMT must be identified.

Fu-Fang-Jin-Qian-Cao (FFJQC) is composed of four crude drugs, *Desmodium styracifolium* (Osb.) Merr.(Leguminosae, Guang Jin Qian Cao), *Plantago asiatica* (L.)(Plantaginaceae, Che Qian Cao), *Pyrrosia calvata* (Bak.)Ching (Polypodiaceae, Guang Shi Wei), and *Zea mays* (Linn.) (Gramineae, Yu Mi Xu), in the ratio of 4:2:2:1, and has been used as a Chinese herbal formula to treat urolithiasis for many years. DS, which is the key pharmacological component of FFJQC, has been confirmed to reduce CaOx deposition in the kidneys and alleviate crystal-mediated damage through its anti-inflammatory and antioxidant functions (Rodgers et al. 2014; Xiang et al. 2015). As shown in our previous study, DS attenuates renal injury and CaOx induced oxidative damage by inhibiting the increased autophagic activity and renal fibrosis (Hou et al. 2018). However, the mechanisms underlying the effects of FFJQC on the CaOx induced renal EMT have not been determined, but are explored in present study.

## Materials and methods

### Chemicals and reagents

Glyoxylic acid was purchased from Tokyo Chemical Industry (TCI, Tokyo, Japan). FFJQC was obtained from Vantone Pharmaceutical (Guangxi, China). The representative HPLC chromatograms of FFJQC and their reference standards are provided in Figure S1. Based on the comparisons with the retention times of standard compounds, mangiferin and schaftoside were identified as the two major components of FFJQC. The result demonstrated that all batches of FFJQC meet the Chinese Pharmacopoeia standard and can be used in the following set of experiments (Chen et al. 2019). The cystone^®^ (Himalayas, India) powders were dissolved in saline at a concentration of 72 mg/mL.

**Figure 1.**
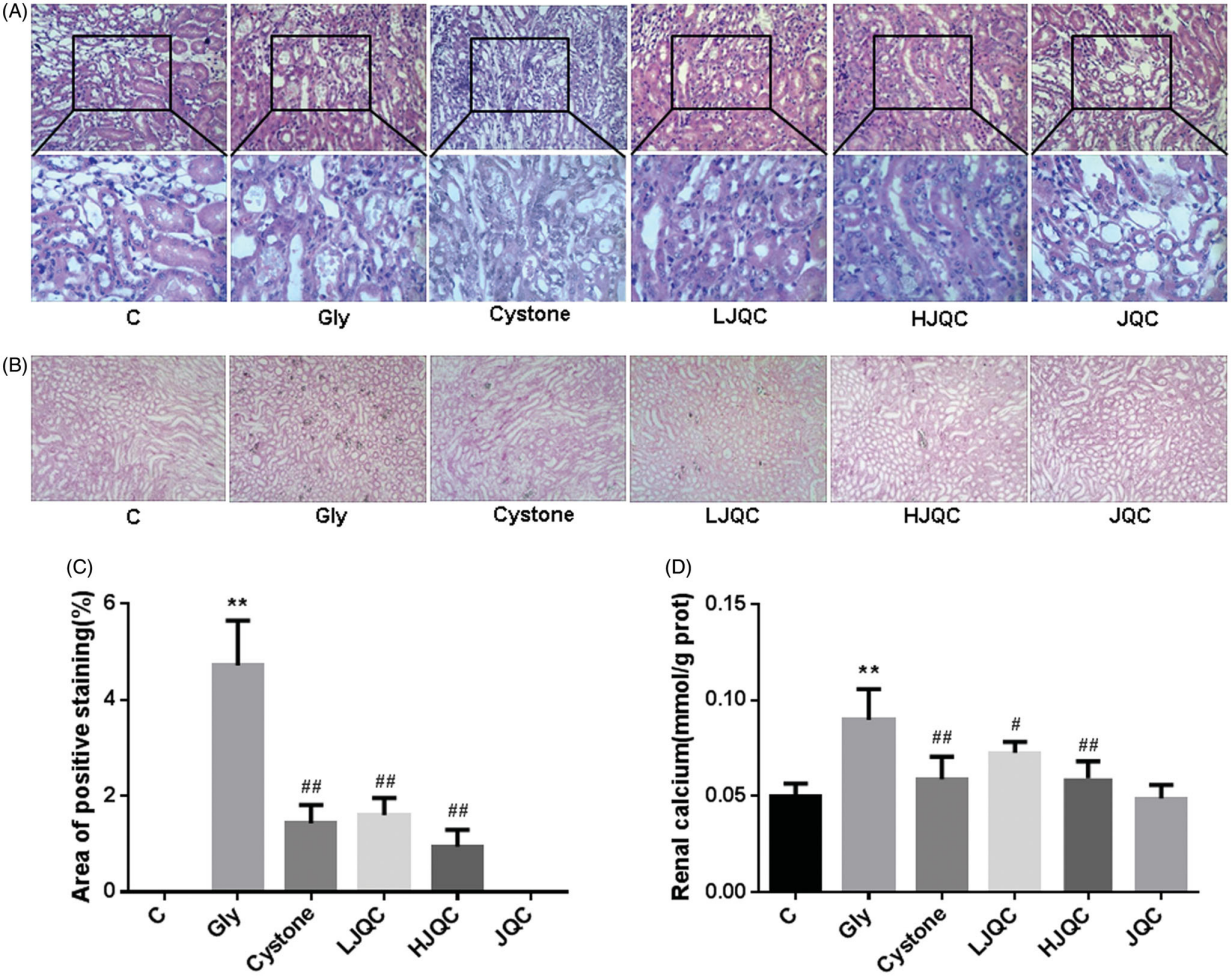
FFJQC inhibits crystal formation in the kidney and ameliorates kidney injury. (A) Representative images of HE staining of the kidney cortex and medulla junction. (B) Representative photomicrographs of von Kossa staining for calcium deposition. (C) Semiquantitative analysis of calcium deposition in the areas displaying positive staining from 20 randomly selected fields of view. (D) Renal calcium levels were determined. Control group (C), glyoxylate-induced calcium oxalate group (Gly), glyoxylate and cystone-treated group (Cystone), glyoxylate and low-dose FFJQC treatment group (LJQC), glyoxylate and high-dose FFJQC treatment group (HJQC), and FFJQC treatment group (JQC). **p < 0.01 Gly group vs. C group; #*p* < 0.05 and ##*p* < 0.01 Cystone/LJQC/HJQC groups vs. Gly group. All data are presented as means ± standard deviations.

Rabbit polyclonal antibodies against cytokeratin 18 (CK18, PAB231Mu01), TGF-β1 (PAA124Mu01), Collagen II (COL2, PAA572Mu01), TGF-β RI (PAA397Mu01) and TGF-β RII (PAC972Mu01) were purchased from CLOUD-CLONE CORP. (Wuhan, China). Rabbit monoclonal antibodies against clusters of EGF-like modules containing mucin-like hormone receptor-like 1 (F4/80, #70076S), E-cadherin (#3195), vimentin (#5741S), α-SMA (#19245) and mouse monoclonal antibodies against GAPDH (#51332) and β-actin (#3700) were purchased from Cell Signalling Technology (CST, MA, USA). Rabbit monoclonal antibodies against pSmad2 (ab188334) and pSmad3 (ab52903) and a rabbit polyclonal antibody against Smad7 (ab216428) were obtained from Abcam (Cambridge, UK). A mouse anti-rabbit IgG-HRP secondary antibody for immunohistochemical staining was purchased from Santa Cruz Biotechnology, Inc. (CA, USA). A Cy3 Affinipure goat anti-rabbit IgG (H + L) for immunofluorescence staining was purchased from Jackson ImmunoResearch Laboratories (NJ, USA). IRDye 800CW goat anti-mouse IgG (H + L) and IRDye 800CW goat anti-rabbit IgG (H + L) antibodies for Western blotting were obtained from LI-COR Biotechnology (NE, USA).

### Animal experiments and sample collection

60 male C57BL/6 mice (8 weeks old) weighing 22-26 g were included in this experiment and purchased from Shanghai SLAC Lab Animal Co., Ltd. (Shanghai, China). All animal studies were performed in accordance with the National Institutes of Health (NIH) Guide for the Care and Use of Laboratory Animals. The mice were housed in an animal room with a 12 h light/dark cycle and were randomly divided into 6 experimental groups (*n* = 10) as follows: control group (C), glyoxylate-induced CaOx group (Gly), glyoxylate and cystone treatment group (Cystone), glyoxylate and low-dose FFJQC treatment group (LJQC), glyoxylate and high-dose FFJQC treatment group (HJQC), and FFJQC treatment group (JQC).

To establish the CaOx crystal renal injury model, mice were intraperitoneally (i.p.) injected with 100 mg/kg glyoxylate once daily for 6 days using previously reported experimental methods to establish the CaOx crystal-induced renal injury model (Okada et al. 2007; Taguchi et al. 2014). FFJQC was dissolved in a saline solution at a concentration of 270 mg/mL, and the mice were intragastrically (i.g.) administered FFJQC at a dose of 1.35 (low dose) or 2.7 (high dose) g/kg body weight according to the human-mouse dose conversion. The mice in the cystone group were i.g. administered 1.2 g/kg Cystone^®^, which served as a positive reference control. The mice in the Gly, Cystone, LJQC and HJQC groups were i.p. injected with glyoxylate once daily for 6 days, while the mice in the C and JQC groups were i.p. injected with a similar volume of normal saline. Four hours after the i.p. glyoxylate/saline injection, the mice in the LJQC/HJQC/JQC groups were i.g. administered low/high/low doses of FFJQC respectively; the Cystone group was i.g. administered 1.2 g/kg cystone^®^ once daily for 6 days, while the C and Gly groups were i.g. administered a similar volume of normal saline.

At the end of the experiment, the mice were all anaesthetised with sodium thiopental. After *in situ* transcardial perfusion, the right kidneys were immediately removed and stored at −80 °C until biochemical analysis. The left kidneys were fixed with 10% buffered formalin for pathological analysis.

### Histopathological analysis

Kidney samples were embedded in paraffin and sectioned at a thickness of 3 μm for histopathological examinations. Sections were stained with hematoxylin-eosin (HE) and reviewed by an independent pathologist, and 20 images captured at magnifications of 200× and 400× were randomly selected from every mouse. The severity of tubular changes in each image was examined and classified as severe (3 points), moderate (2 points), mild (1 point), and negative (0 points).

For von Kossa staining, sections underwent deparaffinization and hydration using a series of dilutions of xylene and alcohol, followed by staining with a von Kossa kit from Jiemei Gene (Shanghai, China) and subsequent eosin counterstaining from Beyotime Institute of Biotechnology (Jiangsu, China). Then, the stained slices were assessed under a microscope (Nikon Eclipse 50i; Nikon Corporation., Tokyo, Japan) to determine the distribution of black CaOx crystal deposits. The number of crystals in a total cross-sectional tissue area from 20 randomly selected fields (200× magnification) was determined using Adobe Photoshop software version 7.0 from Adobe Systems, Inc. (CA, USA).

Immunohistochemical staining was performed using a diaminobenzidine (DAB) kit from Boster (Hubei, China). Positive staining for CK18, F4/80, TGF-β RI and TGF-β RII was measured as the ratio of the integrated optical density/field of kidney cross-sections using Image-Pro Plus software version 6.0. Immunofluorescence staining was employed to determine the levels of the E-cadherin protein. A semiquantitative analysis of CK18, F4/80, TGF-β RI, TGF-β RII and E-cadherin staining was conducted using Image-Pro Plus 6.0 software (Media Cybernetics, MD, USA), and 20 fields of view (200× magnification) from each group were analyzed.

### Western blot analysis

Total proteins were extracted from the corticomedullary tissues of mouse kidneys using Whole Protein Lysis Buffer from KeyGEN Biotech (Jiangsu, China). A BCA Protein Assay Kit from Thermo Fisher Scientific (MA, USA) was used to evaluate the concentrations of soluble proteins. A 10% SDS-PAGE gel from BBI Life Sciences (Shanghai, China) was used to separate proteins that were then transferred onto a nitrocellulose blotting (NC) membrane from GE Healthcare Life Sciences (Little Chalfont, UK). Then, 5% bovine serum albumin (BSA) was used to block the NC membrane at room temperature for 2 h. Afterwards, the NC membrane was incubated with specific primary antibodies at 4 °C overnight. The NC membrane was washed with Tris-buffered saline containing Tween-20 (TBST) 3 times and incubated with fluorescent dye-conjugated secondary antibodies at room temperature for 2 h in the dark. The fluorescence intensity was detected with an Odyssey infra-red fluorescence scanner from LI-COR Biotechnology (NE, USA). This experiment was repeated at least 3 times. The primary antibodies included E-cadherin (1:1000), vimentin (1:1000), α-SMA (1:1000), pSmad2 (1:1000), pSmad3 (1:1500), Smad7 (1:500), TGF-β RI (1:800), TGF-β RII (1:800), β-actin (1:5000), and GAPDH (1:5000). The secondary antibodies included IRDye 800CW goat anti-rabbit IgG (H + L) (1:10000) and IRDye 800CW goat anti-mouse IgG (H + L) (1:10000).

### Data processing and statistical analysis

Data are presented as means ± standard deviations (SD). Histogram and statistics were generated using GraphPad Prism software. The statistical significance of differences in the mean values was determined using one-way ANOVA and Tukey’s *post hoc* test with SPSS 17.0 software from IBM (NY, USA). Differences were considered significant when the *p* value was less than 0.05.

## Results

### FFJQC inhibit the formation of CaOx crystals and alleviate renal injury

Representative photomicrographs of the HE- and von Kossa-stained mouse kidney tissues are shown in Figure 1. Kidney tissues from the Gly group showed severe tubular dilation, tubular atrophy, and a widened interstitial space with severe inflammatory cell infiltration. At low magnification, visible crystals were observed in the whole kidney, mainly at the junction of the cortex and medulla. At high magnification, we observed crystals at the surface/cytoplasm of the epithelial cells and crystal accumulation in the renal tubule lumen. As the crystals aggregated, the tubular damage gradually increased. The administration of cystone and the FFJQC extract significantly attenuated tubulointerstitial damage (Figure 1(A)). After the intraperitoneal injection of glyoxylate, the CaOx crystals formed in the tubular lumens, as shown clearly by von Kossa staining, and the number of crystals located between the renal medulla and the cortex was obviously reduced by LJQC (1/3) and HJQC (1/5) treatments. The therapeutic effect of FFJQC on the formation of calcium oxalate crystals was similar to that of cystone (Figure 1(B,C)). The calcium content of the renal tissue showed the same trend, as shown in Figure 1(D). The JQC group was not different from the control group in terms of the HE and von Kossa staining and the calcium content of the renal tissue (Figure 1).

### FFJQC regulate the CaOx-induced renal EMT

**Figure 2.**
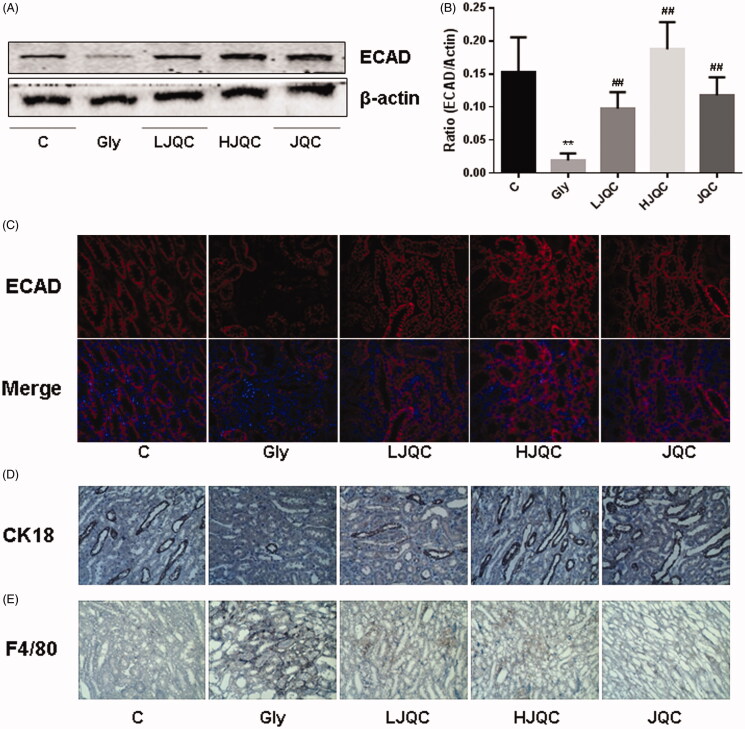
FFJQC inhibits the CaOx-induced renal tubular EMT. Representative images Western blots (A) and immunofluorescence (C) staining for E-cadherin. (B) Ratio of the E-cadherin intensity. Representative images of immunohistochemical staining for CK18 (D) and F4/80 (E). **p < 0.01 Gly group vs. Ctrl group; ^##^*p* < 0.01 LJQC/HJQC groups vs. Gly group. All data are presented as means ± standard deviations.

**Figure 3.**
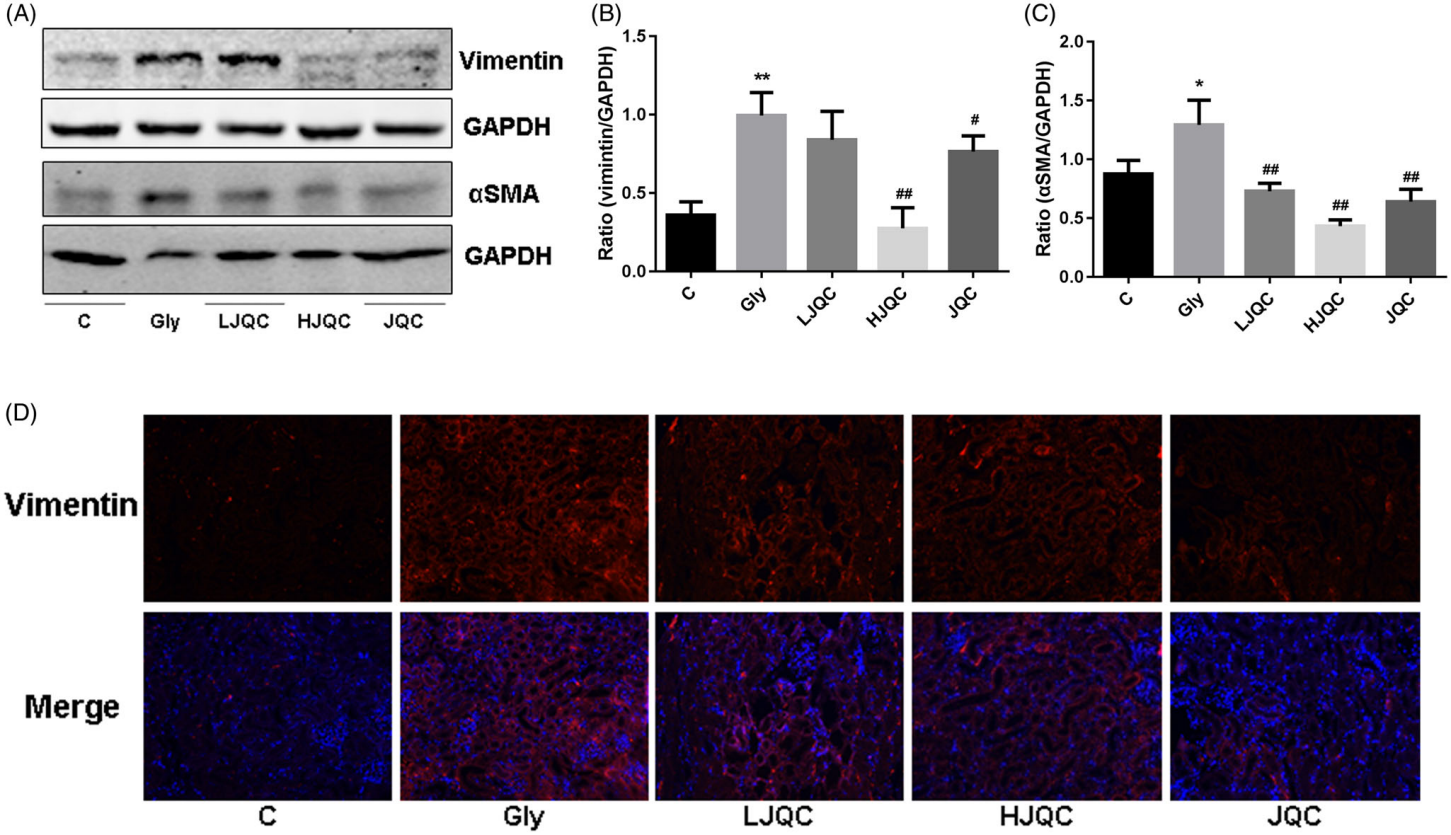
(A) Representative images of the Western blots and analysis of vimentin and α-SMA levels. Ratios of vimentin (B) and α-SMA (C) intensity. (D) Representative images of immunofluorescence staining for vimentin. *p < 0.05 and **p < 0.01 Gly group vs. Ctrl group; #*p* < 0.05 and ##*p* < 0.01 LJQC/HJQC groups vs. Gly group. All data are presented as means ± standard deviations.

The EMT is a major pathogenic factor contributing to the progression of renal fibrosis. The levels of E-cadherin, CK18, α-SMA and vimentin in mice were detected using Western blotting, immunohistochemistry and immunofluorescence staining. Western blotting and immunofluorescence staining revealed decreased E-cadherin expression in the Gly group compared with the C group, and the trends were reversed by the FFJQC treatment (Figure 2(A–C)). Immunohistochemistry showed a similar trend for CK18 expression to E-cadherin (Figure 2(D)). Western blotting and immunofluorescence staining revealed increased levels of the F4/80, α-SMA and vimentin proteins in the Gly group compared with the C group, and the changes were also reversed by the FFJQC treatment (Figures 2(E) and 3(A–D)). Based on these results, the protein levels of epithelial biomarkers decreased and the levels of interstitial biomarkers increased in the Gly group, changes that were reversed by the FFJQC treatment. The JQC group showed no difference from the control group in the levels of EMT-related markers (Figures 2 and 3).

### FFJQC alleviate renal fibrosis mediated by inactive TGF-β-smad pathway

**Figure 4.**
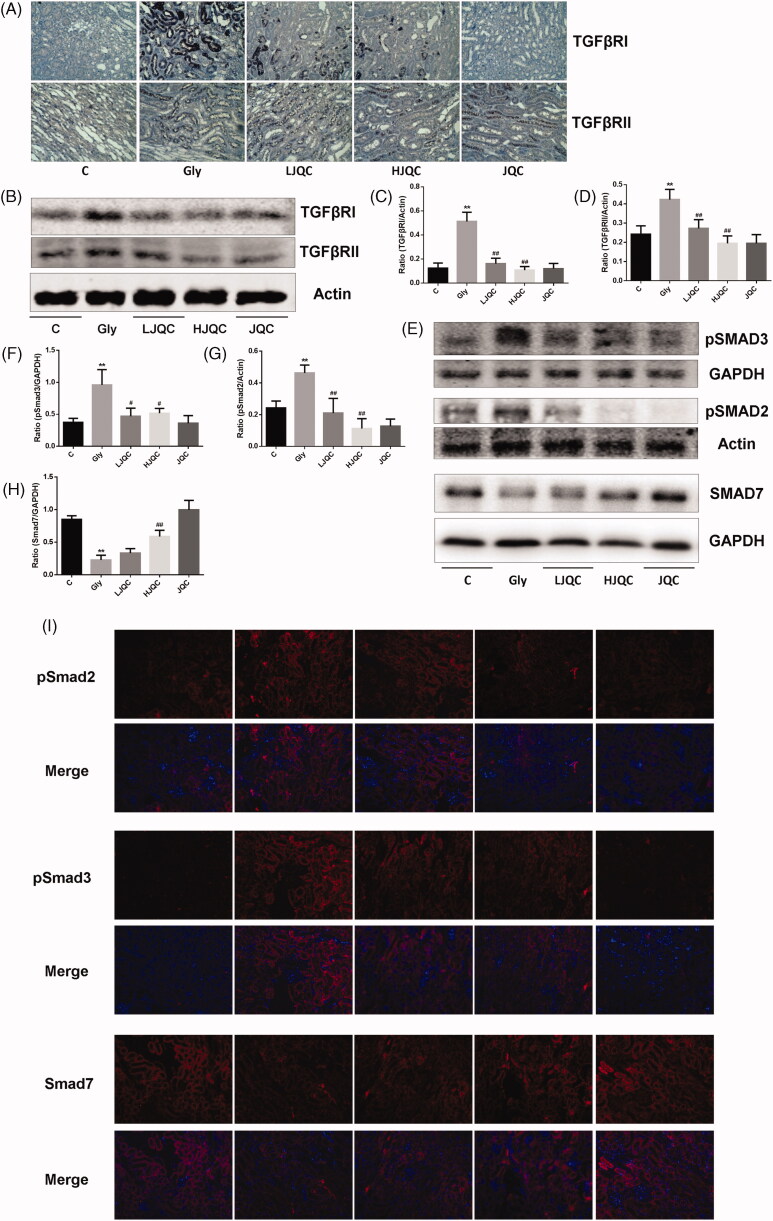
FFJQC inhibits the CaOx-induced activation of the TGF-β-Smad pathway in renal tubules. Representative images of immunohistochemical staining **(**A) and Western blots (B) for TGF-β RI and TGF-β RII expression. Ratios of TGF-β RI (C) and TGF-β RII (D) intensity. Representative images of the Western blots (E) and immunofluorescence staining (I) for pSmad3, pSmad2 and Smad7. Ratios of pSmad3 (F), pSmad2 (G) and Smad7 (H) intensities. **p < 0.01 Gly group vs. Ctrl group; #*p* < 0.05 and ##*p* < 0.01 LJQC/HJQC groups vs. Gly group. All data are presented as means ± standard deviations.

**Figure 5.**
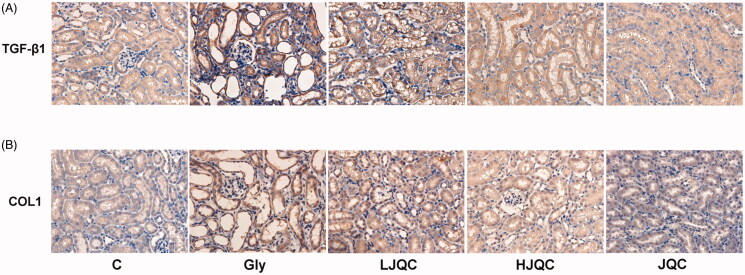
FFJQC inhibits CaOx-induced expression of TGF-β1 and COL2 in renal tubules. Representative images of TGF-β1 (A) and COL2 (B) immunohistochemical staining of mouse kidney tissue in each group.

TGF-β is the most important cytokine involved in the EMT and fibrosis in a variety of animal models. Western blotting and immunohistochemistry showed that the TGF-β RI protein was expressed at high levels in tubular and interstitial cells in mouse kidneys from the Gly group compared with the C group, and the changes were completely reversed by the FFJQC treatment (Figure 4(A–C)). The difference in the expression of TGF-β RII in the Gly group compared with the C group was not as distinct as the difference in TGF-β RI expression, but was still noticeable, as shown in the graph (Figure 4(D)). Furthermore, TGF-β RII levels were also reduced by FFJQC therapy (Figure 4(A,B,D)). Immunohistochemistry of mouse kidney tissue showed that CaOx induced an increase in the expression of TGF-β1 in Gly group, and FFJQC could reduce its expression (Figure 5(A)). In addition, FFJQC could effectively inhibit the accumulation of fibrosis marker COL2 induced by CaOx (Figure 5(B)).

As shown in Figure 4(E–I), Western blotting and immunofluorescence staining revealed significant increases in p-Smad2 and p-Smad3 levels in kidney tissues from the mice in the Gly group, while Smad7 levels were decreased. LJQC/HJQC treatments attenuated the increase in p-Smad2 and p-Smad3 levels, and the HJQC treatment further enhanced the decrease in Smad7 expression. These findings all highlight the activation of the TGF-β/Smad signalling pathway in the Gly group, which was reversed by FFJQC therapy. The JQC group showed no difference in the activation of the TGF-β/Smad pathway compared with the control group (Figure 4).

## Discussion

Crystals can trigger a wide range of kidney injuries, leading to acute kidney injury, CKD or renal colic (Mulay and Anders 2017). CKD has a high incidence and a poor prognosis, and eventually progresses to ESRD. Currently, CKD is an important disease that threatens human health and imposes a substantial economic burden. Kidney fibrosis is an unavoidable consequence of CKD and is characterised by the EMT of tubular epithelial cells (TECs) (Lovisa et al. 2015). According to most previous studies, the EMT is closely related to the occurrence and development of tumours, but in benign diseases, studies on EMT are relatively rare. Therefore, we explored the EMT in the oxalate crystal-induced renal injury.

The EMT refers to the phenotypic conversion of epithelial cells to a fibroblast-like morphology. During this process, cells gradually lose the expression of epithelial markers, such as E-cadherin and cytokeratins, and the expression of mesenchymal markers, such as vimentin and α-SMA, is induced. Cytokeratins are essential for maintaining the structural integrity of the renal epithelium (Okada et al. 2000). In the present study, the levels of E-cadherin and CK18 were reduced and the levels of vimentin and α-SMA were increased in the oxalate crystal-induced renal injury model, indicating that the EMT occurred during the process of crystal formation. F4/80 is a macrophage marker, while macrophages can secrete a number of proinflammatory cytokines, inducing EMT in kidney cells (Liu 2004).

Fibrosis is primarily driven by inflammatory cytokines, including members of the TGF-β superfamily (Meng, Nikolic-Paterson, et al. 2016). TGF-β is also an EMT effector, Smad2/3 and Smad7 are EMT-related signalling molecules. TGF-β is the main driver of fibrosis in subjects with CKD. Inhibition of the TGF-β isoform TGF-β1 or its downstream signalling pathways substantially limits renal fibrosis in a wide range of disease models, whereas TGF-β1 overexpression induces renal fibrosis. In the context of renal fibrosis, Smad2 and Smad3 are activated in both humans and experimental animals with CKD induced by obstructive kidney disease (Zhou et al. 2017). Smad7 is a negative feedback regulator of the TGF-β1/Smad pathway and protects against TGF-β1-mediated fibrosis by inducing receptor degradation to block the recruitment and phosphorylation of Smad2 and Smad3 (Heldin and Moustakas 2012).

FFJQC consists of *D. styracifolium*, *P. asiatica*, *P. calvata,* and *Z. mays*. Each herb has been studied to varying degrees (Huang et al. 2014; Kho et al. 2017; Hou et al. 2018; Pardede and Muchlisyam 2018). In our previous study (Chen et al. 2019), we focussed on the serum metabolic profile of FFJQC, and in the present study, we focussed on its mechanism of action on fibrosis on the kidney. We systematically summarised the active ingredients reported in previous studies and found that some exert therapeutic effects on the EMT and fibrosis with varying degrees and mechanisms. Among these components, quercetin (Lu et al. 2015) and mangiferin (Zhu et al. 2015) inhibit renal tubular EMT and fibrosis in subjects with diabetic nephropathy, and apigenin antagonises renal fibrosis in subjects with hypertension (Wei et al. 2017). Ferulic acid (Meng et al. 2011), luteolin (Kim et al. 2016), rutin (Wang et al. 2016), quercetin and hyperoside (Yan et al. 2014) inhibit renal tubulointerstitial fibrosis in rats with obstructive nephropathy. Liquiritigenin ameliorates damage in rats with chronic renal failure by inhibiting fibrotic responses (Zhang, Zhu, et al. 2017), while ursolic acid reduces renal fibrosis in rats with CKD (Thakur et al. 2018). Wogonin (Meng, Ren, et al. 2016) and succinic acid (Faust et al. 2009) relieve renal fibrosis in renal TECs. In our study, the TGF-β/Smad pathway was activated in response to oxalate crystal-induced renal injury and was reversed by FFJQC therapy, indicating that the protective effect of FFJQC on crystal-induced kidney injury may be achieved by inhibiting the TGF-β/Smad pathway.

## Supplementary Material

Figure S1
