# Supplementary figures and images for: Fu-Fang-Jin-Qian-Cao herbal granules protect against the calcium oxalate-induced renal EMT by inhibiting the TGF-β/smad pathway
Source: Pharm Biol. 2020 Nov 15;58(1):1124–31. doi: 10.1080/13880209.2020.1844241 (PMC7671650; doi:10.1080/13880209.2020.1844241)

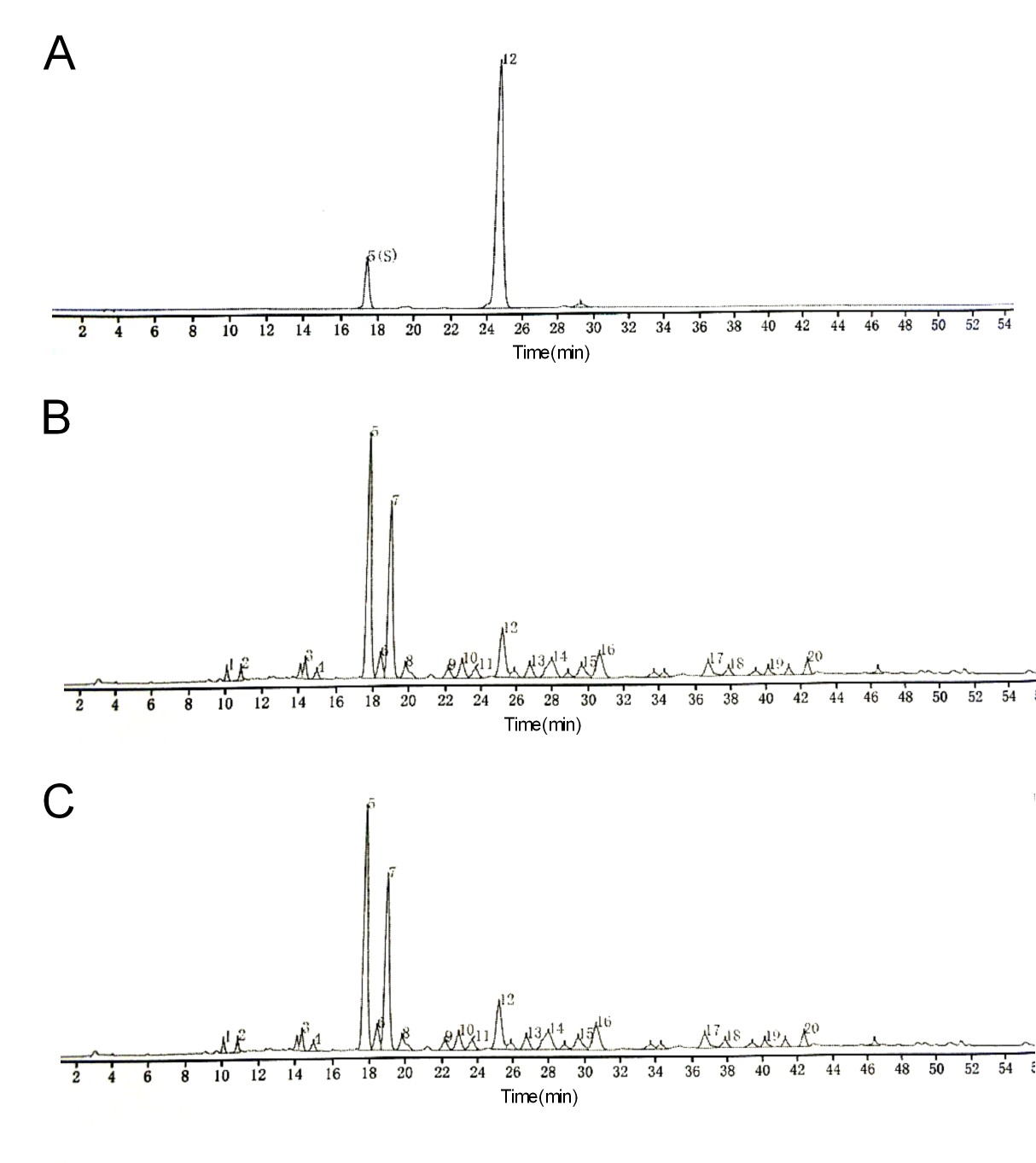

Supplement: Figure S1 [file IPHB_A_1844241_SM4940.jpg]
